# Doxorubicin-Loaded PEG-PCL-PEG Micelle Using Xenograft Model of Nude Mice: Effect of Multiple Administration of Micelle on the Suppression of Human Breast Cancer

**DOI:** 10.3390/cancers3010061

**Published:** 2010-12-28

**Authors:** Nguyen-Van Cuong, Jian-Lin Jiang, Yu-Lun Li, Jim-Ray Chen, Shyh-Chuan Jwo, Ming-Fa Hsieh

**Affiliations:** 1 Department of Biomedical Engineering, Chung Yuan Christian University, 200, Chung Pei Rd., Chung Li, Taiwan; E-Mail: nvc2505@gmail.com (N.V.C.); akito2000.tw@yahoo.com.tw (J.L.J.); lun1126123@hotmail.com (Y.L.L.); 2 Department of Chemical Engineering, Ho Chi Minh City University of Industry, 12 Nguyen Van Bao St, Ho Chi Minh, Vietnam; E-Mail: nvc2505@gmail.com (N.V.C.); 3 Department of Pathology, Chang Gung Memorial Hospital at Keelung, Taiwan and Chang Gung University, College of Medicine, Taoyuan, Taiwan; E-Mail: jimrchen@cgmh.org.tw (J.R.C.); 4 Division of General Surgery, Chang Gung Memorial Hospital at Keelung, Taiwan and Chang Gung University, College of Medicine, Taoyuan, Taiwan; E-Mail: jowsc@adm.cgmh.org.tw (S.C.J.)

**Keywords:** nanoparticles, drug delivery system, monomethoxy poly(ethylene glycol), poly(ε-caprolactone), safety evaluation, biodistribution, antitumor activity

## Abstract

The triblock copolymer is composed of two identical hydrophilic segments Monomethoxy poly(ethylene glycol) (mPEG) and one hydrophobic segment poly(ε-caprolactone) (PCL); which is synthesized by coupling of mPEG-PCL-OH and mPEG-COOH in a mild condition using dicyclohexylcarbodiimide and 4-dimethylamino pyridine. The amphiphilic block copolymer can self-assemble into nanoscopic micelles to accommodate doxorubixin (DOX) in the hydrophobic core. The physicochemical properties and *in vitro* tests, including cytotoxicity of the micelles, have been characterized in our previous study. In this study, DOX was encapsulated into micelles with a drug loading content of 8.5%. Confocal microscopy indicated that DOX was internalized into the cytoplasm via endocystosis. A dose-finding scheme of the polymeric micelle (placebo) showed a safe dose of PEG-PCL-PEG micelles was 71.4 mg/kg in mice. Importantly, the circulation time of DOX-loaded micelles in the plasma significantly increased compared to that of free DOX in rats. A biodistribution study displayed that plasma extravasation of DOX in liver and spleen occurred in the first four hours. Lastly, the tumor growth of human breast cancer cells in nude mice was suppressed by multiple injections (5 mg/kg, three times daily on day 0, 7 and 14) of DOX-loaded micelles as compared to multiple administrations of free DOX.

## 1. Introduction

Doxorubicin (DOX), an anthracycline drug has proven very effective for the treatment of breast, ovarian, prostate, brain, cervix and lung cancers [1-4]. Nevertheless, drawbacks such as cardiac toxicity, short half-life and low solubility in aqueous solution have hindered its application. In addition, some tumor cells showed multidrug resistance (MDR), which has been attributed to the P-glycoprotein (P-gp) efflux pump on the plasma membrane [5,6]. To overcome these obstacles, strategies such as encapsulating DOX into the core of polymeric nanoparticles by chemical conjugation or physical entrapment have been attempted [7-11]. The employment of polymeric nanoparticles also provides an opportunity to tailor the release profiles of encapsulated drugs [12,13].

In recent years, biodegradable polymeric nanoparticles have been extensively employed as effective drug delivery systems to enhance the efficacy and safety of encapsulated drugs. Nanoparticle carriers provide a better accumulation in tumor tissues through an enhanced permeability and retention (EPR) effect [14,15], ability to overcome multidrug resistance [11], and better pharmacokinetics of drug *in vivo* [16]. Nanoparticles composed of poly(ε-caprolactone) (PCL) and poly(ethylene glycol) (PEG) have been demonstrated as great potential carriers for the delivery of anti-cancer agents. PCL is a biodegradable, biocompatible and nontoxic thermoplastic polyester [17]. PEG is a common constituent for the hydrophilic outer shell and is known to reduce the adhesion of plasma proteins, solubility in water and organic solvents, stabilization of particles and lack of toxicity [4,18]. Additionally, polymeric nanoparticles with hydrophilic PEG outer shell can potentially increase the circulation time of drugs and can prevent recognition by macrophages of the reticuloendothelial system (RES) after intravenous injection [18-20]. Recently, amphiphilic triblock copolymers consisting of poly (ethylene glycol) as hydrophilic segment, and poly (*ε*-caprolactone) as hydrophobic block, have been used as drug delivery systems, such as 4′-demethyl-epipodophyllotoxin [21], honokiol [22], folic acid [23], nimodipine [24] and doxorubicin [25,26].

Previously, we reported the preparation of a triblock copolymeric (poly(ethylene glycol)/poly(ε-caprolactone)/poly(ethylene glycol)) micelle (PEG-PCL-PEG) aiming for its application as a nanocarrier for hydrophobic drugs [27]. A hydrophobic drug such as doxorubicin was successfully loaded into triblock copolymeric micelle that was prepared by the self-assembly method in an aqueous solution. The release profile and cellular uptake of DOX and its cytotoxic effects against drug-sensitive (MCF-7) breast cancer cell lines were also investigated. General prerequisites for a new drug delivery system should be that it is non-cytotoxic, biocompatible, and in most cases, biodegradable. Therefore, safety evaluation of triblock copolymeric micelle including the *in vitro* and *in vivo* toxicological studies is required before further application of triblock copolymeric micelles in biomedical fields. In this work, we evaluated the safety of prepared PEG-PCL-PEG micelle as an intravenous drug delivery system in a series of tests including cytotoxicity tests, *in vitro* nitric oxide production and hemolytic tests and *in vivo* acute toxicity tests in ICR mice. Furthermore, the biodistribution of DOX-loaded micelle was evaluated *in vivo.* Importantly, the therapeutic potential of single and multiple administrations of DOX-loaded micelle were investigated using the nude mice xenograft model.

## 2. Results and Discussion

### 2.1. Physicochemical Properties of DOX-Loaded PEG-PCL-PEG Micelle

The amphiphilic triblock copolymer built with hydrophilic mPEG segments and hydrophobic PCL segment and by a mild coupling agent-DCC/DMAP was prepared as previously reported [27]. The triblock copolymer was characterized by proton nuclear magnetic resonance (^1^H NMR), gel permeation chromatography (GPC), Fourier transform infrared spectroscopy (FT-IR), X-ray diffraction (XRD) and differential scanning calorimetry (DSC). The molecular weight of triblock copolymer (PEG-PCL-PEG) was 31,000 kD by ^1^H NMR [27]. The amphiphilic triblock copolymer can self assemble to form a core/shell structure in an aqueous solution. The shell and the inner core of micelle are composed of the hydrophilic PEG polymer and the hydrophobic PCL polymer, respectively. The critical micelle concentration (CMC) value of the triblock copolymers is 5.1 × 10^−4^ mg/mL. This copolymer contains the hydrophobic segment, enabling the encapsulation of the hydrophobic drug in the core of micelle. As a well-known anti-cancer reagent, DOX is limited by its acute toxicity of free drugs to normal tissues, low water solubility and inherent multi-drug resistance effects. In an attempt to overcome the disadvantages of toxicity and drug-resistance and increase selectivity towards cancer cells, the hydrophobic DOX was physically entrapped in the core of micelle. The drug loading efficiency (DLE) and drug loading content (DLC) were calculated in a range of 55.4 to 57% and 5.4 to 8.5%, respectively (Table 1).

### 2.2. Stealth Property of PEG-PCL-PEG Micelle: The Response of Macrophage Cells toward the PEG-PCL-PEG Micelle

**Figure 1.**
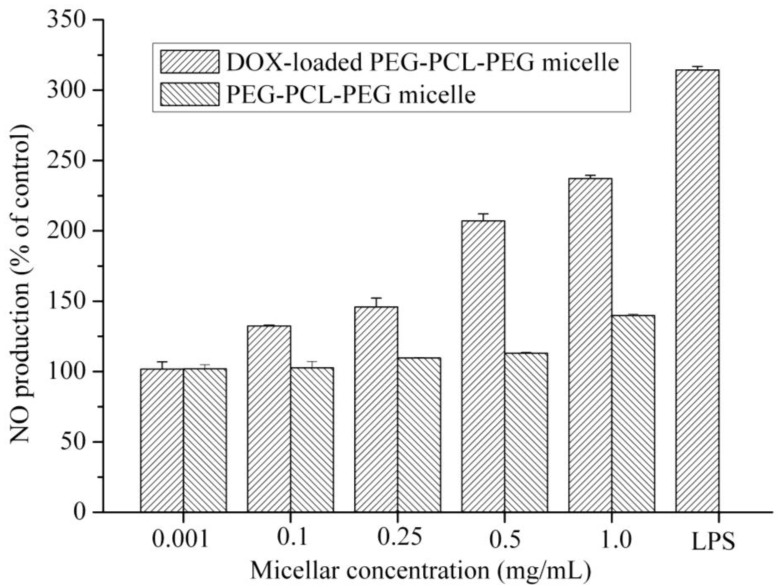
Effects of PEG-PCL-PEG micelle and DOX-loaded PEG-PCL-PEG micelle on the level of nitric oxide in RAW264.7 cells. Data represents the mean ± standard error of the mean of four experiments (*p* < 0.01 is significantly different from the LPS).

Delivery of therapeutic agents by using polymeric nanoparticles can be effectively targeted to specific organs or reduce the drug side effects. However, intravenously injected polymeric nanoparticles are rapidly eliminated from the systemic circulation because of the activation of macrophages of the reticuloendothelial system (RES). Consequently, various inflammatory substances including nitric oxide (NO), cytokines and reactive oxygen species (ROS) are produced. We performed NO and ROS assays to evaluate the *in vitro* cytotoxicity of polymeric micelle on macrophages. The results showed that micelles did not affect NO production up to 0.5 mg/mL (Figure 1). The NO production by micelles was close to that of the control group. In contrast, the lipopolysaccharides (LPS) (100 ng/mL) significantly increased the NO production by macrophage cells. Moreover, DOX-loaded PEG-PCL-PEG micelle exhibited higher NO level than that of PEG-PCL-PEG micelle at concentrations from 0.1 to 1.0 mg/mL (DOX concentrations were 78.5, 39.2 and 7.85 μg/mL for 1.0, 0.5 and 0.1 mg/mL polymeric micelle, respectively.). DOX-loaded PEG-PCL-PEG showed NO production 2-fold higher than that of control. In contrast, NO secretion of PEG-PCL-PEG micelle was increase only 1.13-fold over the control group at a micelle concentration of 0.5 mg/mL.

**Figure 2.**
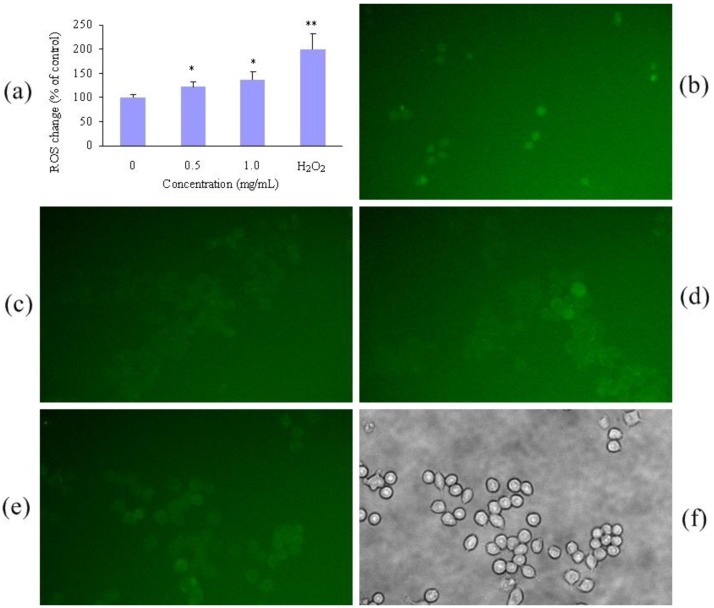
Effects of PEG-PCL-PEG micelle on the level of ROS in RAW264.7 cells. (**a**) Cells were treated with micellar solution (0, 0.5 and 1.0 mg/mL) and H_2_O_2_ solution for 24 h, incubated with 20 μM dichlorofluorescin-diacetate (DCFH-DA) for 30 min, and then washed with phosphate-buffered saline. The cells were lysed with 0.5% Triton in PBS and the fluorescence of aliquot was measured. The bars marked with * and ** (*p* < 0.01) showed statistically significant differences from the treated group compared with control and H_2_O_2_ solution (1 mM), respectively. (**b**–**e**) Cells were observed with a fluorescent microscope (200×): H_2_O_2_ solution (**b**); Control (**c**), 0.5 mg/mL (**d**) and 1.0 mg (**e**). (**f**) The morphology of cells treated with 1.0 mg/mL micelles after 24 h.

Nanoparticles were reported to induce toxicity by ROS-mediated oxidative stress [28]. Therefore, ROS was measured to evaluate the level of oxidative stress in RAW264.7 cells treated with polymeric micelles at 0.5 and 1.0 mg/mL. The fluorescence intensity of dichlorofluorescein (DCF), a permeative indicator of oxidative stress, increased accordingly with increased concentration of polymeric micelle. ROS generation at 1.0 mg/mL was elevated to approximately 130% of the control. However, cells exposed to H_2_O_2_ solution (1 mM) showed approximately 200% ROS generation compared to the control group. Figure 2 shows the representative figures of ROS formation visualized under a fluorescent microscope.

**Figure 3.**
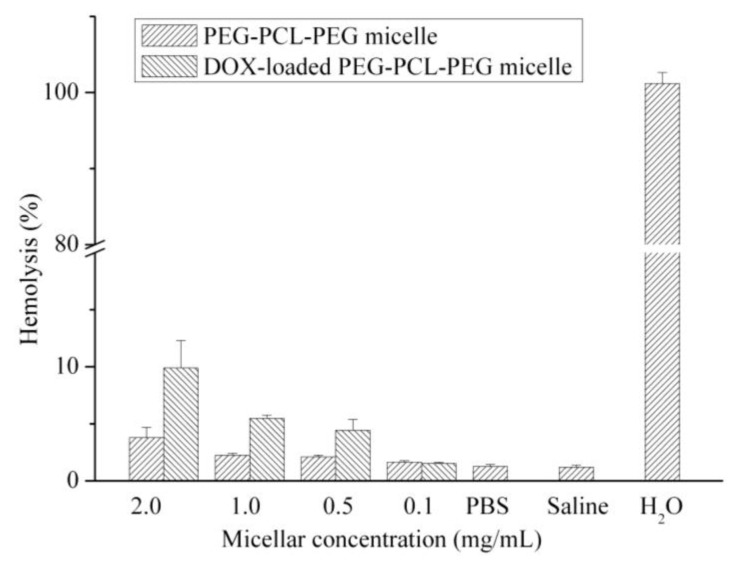
Hemolytic test on PEG-PCL-PEG micelle and DOX-loaded PEG-PCL-PEG micelle. Data represents the mean ± standard error of the mean of three experiments (*p* < 0.01 compared to saline group).

Furthermore, the biocompatibilities of PEG-PCL-PEG micelle and DOX-loaded PEG-PCL-PEG micelle in the presence of red blood cells (RBCs) were examined by a hemolytic test. As shown in Figure 3, the percentage of hemolysis increased accordingly with the concentration of micelle. DOX-unloaded polymeric micelle at a concentration of 2 mg/mL caused a slight increase in hemolysis when compared with that of the negative control (saline solution) and blank solution (PBS buffer). However, DOX-loaded micelle exhibited higher hemolytic activity than that of DOX-unloaded PEG-PCL-PEG micelle. This may be attributed to the release of DOX in medium that caused hemolysis. The results also demonstrated that the hemolytic activity of micellar DOX depended on the DOX concentration. DOX concentrations were 157, 78.5, 39.2 and 7.85 μg/mL for 2.0, 1.0, 0.5 and 0.1 mg/mL polymeric micelle, respectively.

**Figure 4.**
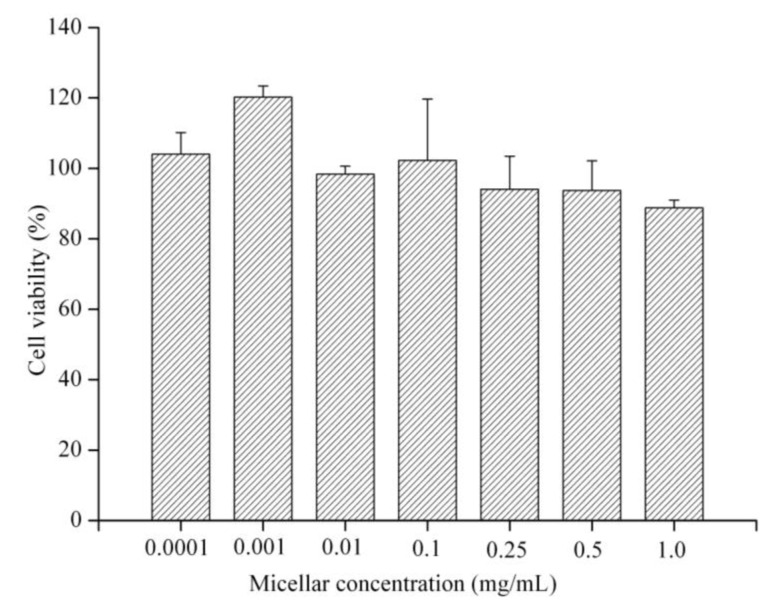
The cytotoxicity of PEG-PCL-PEG micelle against MCF-7 cells. Data represents the mean ± standard error of the mean of four measurements (*p* > 0.4 compared to control).

The cytotoxicity of PEG-PCL-PEG micelle to MCF-7 cells was evaluated using MTT assays. Figure 4 demonstrated the cell viability after 24 h incubation with polymeric micelle of triblock copolymer at various concentrations (0.001, 0.01, 0.1, 0.5 and 1.0 mg/mL). The results showed that cell viability of MCF-7 cell decreased accordingly as the concentration of micelle increased. However, the lowest cell viability approximately 89% was observed at a concentration of 1.0 mg/mL. The cells viability assay indicates that the PEG-PCL-PEG micelle has generally low cytotoxicity to the MCF-7 cells with concentration up to 1.0 mg/mL. The data of macrophage response and hemolysis test suggest that the PEG-PCL-PEG micelle prepared in this study had moderate *in vitro* toxicity and could be safely used for intravenous injection in animal.

### 2.3. In Vitro Cytotoxicity of DOX-Loaded Micelle

**Figure 5.**
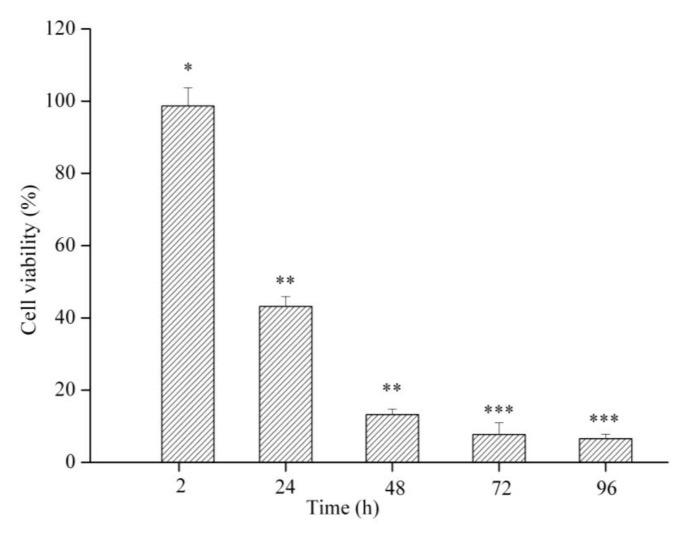
Cytotoxicity of DOX-loaded micelle against MCF-7 cells. The cells were incubated with DOX-loaded micelle (DOX concentration 10 μg/mL) for 2, 24, 48, 72 and 96 h at 37 °C. Each bar represents the mean of five measurements ± S.D. Bar marked with * (*p* = 0.89) showed no significant difference between 2 h incubation and control. Bars marked with ** (*p* < 0.001) showed significant difference between 2 h and 24 h and 48 h incubation. *** *p* > 0.27 as compared to 48 h incubation.

The *in vitro* cytotoxic effect of DOX-loaded micelle was studied using a tetrazolium dye (MTT assay) in MCF-7 cells. The cell viability was determined by incubating cells in 10 μg/mL DOX for different periods of time. Figure 5 shows the cell viability of MCF-7 treated with DOX-loaded micelle. The cell viability decreased significantly with increased time of treatment (2–96 h). The reason that DOX-loaded micelle did not show cytotoxicity after a 2-h incubation could be attributed to the lag phase of DOX. Similar results were obtained with 3-h incubation of DOX in other formulations [29,30]. However, significant cytotoxicity was observed at 24 h and 48 h. As shown in Figure 4, the cell viability decreased from 98.7% at 2 h to 43.2% and 13.2% at 24 h and 48 h, respectively, compared to control. Similarly, the cell viability was 7.7% and 6.6% for 72 h and 96 h, respectively. These results indicate that the cell viability did not significantly decrease when the treatment time was prolonged, which could be attributed to the sustained effect of DOX-loaded micelle and/or loss of cells. From the above results, we confirmed that the optimal treatment time for cell viability assay of DOX-loaded micelle was approximately 48 h, and prolonging the treatment time did not lead to more cell death.

### 2.4. Confocal Image of DOX-Loaded Micelle in MCF-7 Cells

**Figure 6.**
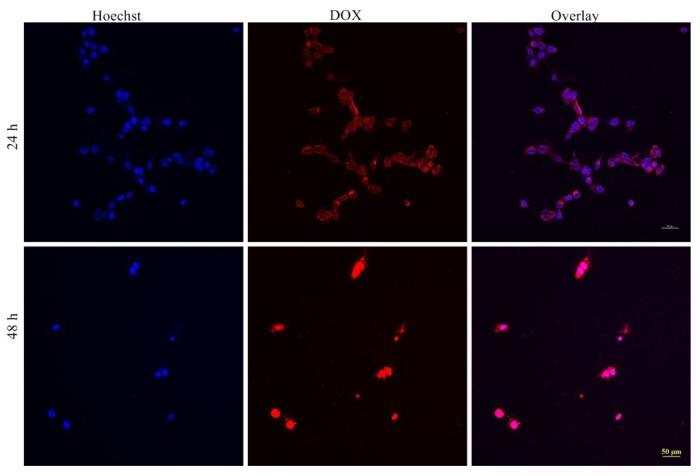
Confocal laser microscopy (CLSM) of MCF-7 cells incubated with DOX-loaded micelle for 24 h and 48 h with DOX concentration of 10 μg/mL (scale bar 50 μm).

Confocal microscopy was employed to visualize the cellular uptake and the internalization of DOX-loaded micelle in MCF-7 cells. As shown in Figure 6, the distribution of DOX in cells was different at 24 and 48 h. Red fluorescence of DOX was found to localize in the nuclei of MCF-7 cells, showing a co-localization of nucleus (blue fluorescence of Hoechst) after 48 h. Additionally, the cell number was relatively low compared with that of 24-hour exposure to DOX-loaded micelle. This is attributed to the cytotoxicity effect of DOX. Conversely, only weak fluorescence was observed in nuclei of cells after 24 h exposure and most of DOX was accumulated outside nuclei of cells. The signal observed in the nuclei was attributed to the release of DOX molecules from the micelles. Additionally, red fluorescence was observed only outside the nuclei of cells after 2 h exposure [27]. Furthermore, when the MCF-7 cells were incubated with free DOX, fluorescence signals were observed only in the nuclei of cells not in the cytoplasm at all time-points [27]. The observation of fluorescence in cytoplasm indicated that the DOX-loaded micelle was internalized by the cells through endocytosis and DOX was distributed in the cytoplasm after escaping from the endosome and/or the lysosome [31,32].

### 2.5. Acute Toxicity of Micelle In Vivo

**Figure 7.**
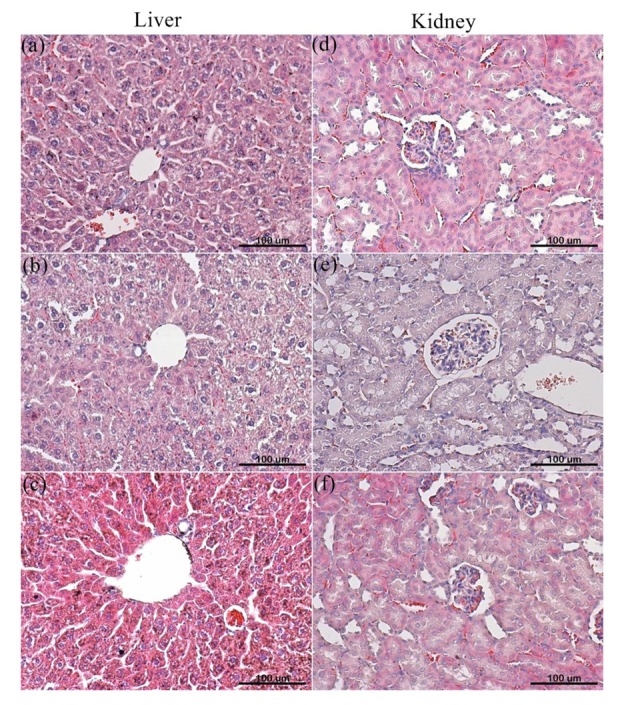
*In vivo* acute toxicity of PEG-PCL-PEG micelle treated mice. Each organ was evaluated by H&E staining after 14 days post-injection. (**a**), (**d**) Control; (**b**), (**e**) Single dose; (**c**), (**f**) multiple doses (scale bar: 100 μm).

The PEG-PCL-PEG micelle was intravenously injected to ICR mice at a dose of 71.4 mg/kg for multiple and single modes for evaluation of possible toxicities of PEG-PCL-PEG micelle. The body weight of mice was not significantly different from control group (injected with PBS) in both single and multiple doses after 14 days. Additionally, no mice died during the whole observation period. Histological examination was performed to evaluate toxicity in kidneys and livers. As the results show in Figure 7, no particular toxicity, no degeneration, necrosis, neutrophils or activation of immunoresponse were observed in liver and kidney when the mice were administered with single or multiple doses. This demonstrated that prepared PEG-PCL-PEG micelle was safe *in vivo* for micellar concentrations of less than 71.4 mg/kg.

### 2.6. Biodistribution of DOX-Loaded Micelle

**Figure 8.**
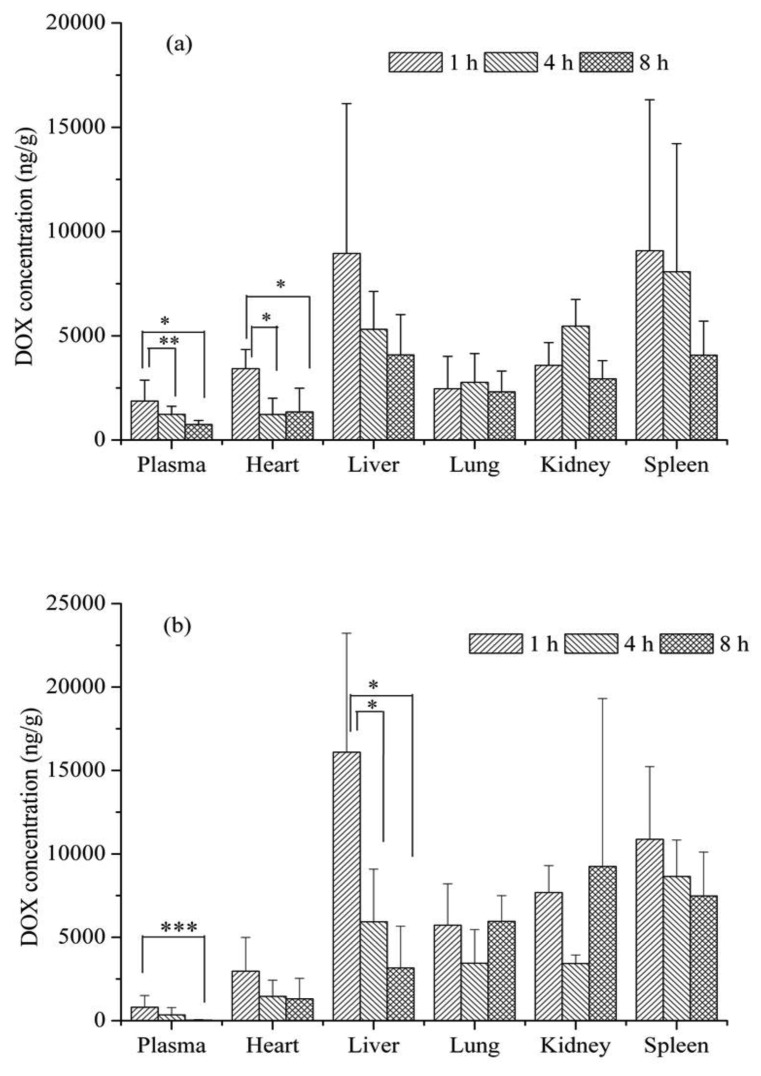
Biodistribution of DOX-loaded micelle (**a**) and free DOX (**b**) after administration at the equivalent 5 mg/kg DOX. Each bar represents the mean of three measurements ± S.D. Bars marked with * (*p* < 0.05) are significantly different between 1 h time-point and 4 and 8 h. Bars marked with ** and *** correspond to *p* values 0.073 and 0.065, respectively.

Biodistribution profile of free DOX and DOX-loaded micelles were examined in Wistar rat. The animal was intravenously administered a dose of 5 mg/kg DOX equivalent. DOX content in plasma, heart, liver, lung, kidney and spleen were measured at three intervals (1, 4 and 8 h). The results indicated that DOX-loaded micelle could prolong the DOX in plasma and exhibited higher DOX concentration in plasma than free DOX (Figure 8). At the 1 and 4 h time-points, the DOX level of DOX-loaded micelle in plasma was 2.3 and 3.6-fold higher than that of free DOX, respectively. Interestingly, at 8 h time-point, the DOX level of free DOX was not detected in plasma, in contrast, it still remained at high concentration in DOX-loaded micelle formulation. Biodistribution patterns to the hearts, lungs, and kidneys did not show substantial accumulation in DOX-loaded micelle and free DOX groups and were not significantly different. The uptake by liver was observed to be higher for free DOX as compared to DOX-loaded micelle at 1 h post-injection.

### 2.7. Antitumor Activity of DOX-Loaded Micelle In Vivo

**Figure 9.**
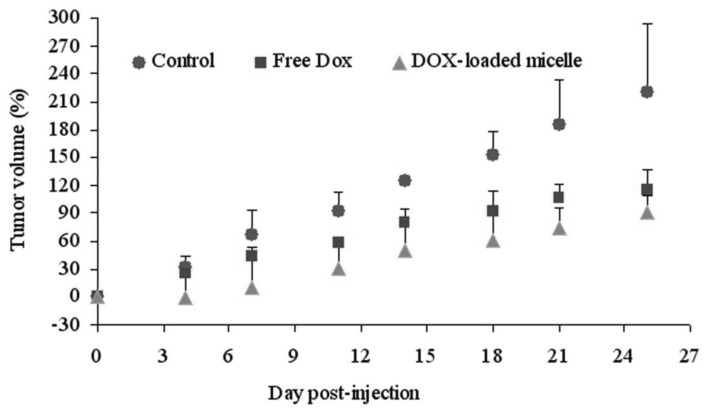
Antitumor effect of free DOX and DOX-loaded micelle in MCF-7 tumor bearing mice. Mice were administered PBS (●) and free DOX (■) and DOX-loaded micelle (▲) *i.v* at the equivalent 5 mg/kg DOX.

The antitumor efficacy of free DOX and DOX-loaded micelles was examined with MCF-7 human breast tumor bearing nude mice. The tumor growth rates of mice treated with free DOX, DOX-loaded micelle and PBS are presented in Figure 9. The free DOX and DOX-loaded micelle exhibited similar effectiveness in preventing tumor growth when mice were administered with single dose (data not shown). However, when the mice were treated with three injections, the DOX-loaded micelle demonstrated the greater growth inhibition of tumor volume in comparison to free DOX. Tumor volumes were decreased up to 60.0% by DOX-loaded micelle and 45.4% by free DOX, compared to that of control group, respectively (Figure 9). Based on the above results, DOX-loaded micelle showed higher tumor targeting efficiency and more therapeutic effects than free DOX. Hence, the hydrophobic drug encapsulated in PEG-PCL-PEG micelle has advantages of prolonged blood circulation, RES uptake prevention, and passive targeting of polymeric micelles to tumor tissue through EPR effect [14,33].

## 3. Experimental Section

### 3.1. Materials

Monomethoxy poly(ethylene glycol) (mPEG, Mn = 5000), ε-caprolactone, doxorubicin hydrochloride (DOX·HCl) and dimethylsulfoxide (DMSO) were purchased from Sigma-Aldrich (St.Louis, MO, U.S.). Stannous 2-ethyl hexanoate (stannous octoate, Sn(Oct)_2_) was obtained from MP Biomedicals, Inc. Methanol, tetrahydrofuran (THF) and acetonitrile (ACN) were HPCL grade and obtained from ECHO chemical (Taiwan).

Human breast cancer cell lines (MCF-7) were kindly provided by Dr. Y.H. Chen of the School of Pharmacy, College of Medicine, National Taiwan University. 3-(4,5-dimethylthiazol-2-yl)-2,5-diphenyl tetrazolium bromide (MTT) was purchased from Sigma-Aldrich (St. Louis, MO, U.S.). Dulbecco's modified eagle's medium (DMEM) and antibiotic/antimycotic were purchased from GIBCO (NY, U.S.). The fetal bovine serum (FBS) was obtained from HyClone (Ultah, U.S.).

### 3.2. Preparation and Characterizations of DOX-loaded PEG-PCL-PEG Micelle

The preparation of PEG-PCL-PEG micelle has been reported elsewhere [27]. The loading of DOX in the micelle was done by first neutralizing 3.0 mg of DOX·HCl with 10 μL TEA in 2.0 mL THF and stirred for 3 h. The resulting solution was added to 20 mg of PEG-PCL-PEG copolymer under stirring. This solution was added to 2.0 mL of double distilled water under stirring for 3 h to form DOX-loaded micelle. To remove un-trapped DOX and TEA, the mixture was next transferred for dialysis against double distilled water for 24 h to produce DOX-loaded micelle (MWCO: 8,000 Da, Spectrum Laboratories, U.S.).

The drug loading efficiency (DLE) was defined as the weight percentage of DOX in micelle relative to the initial feeding amount of DOX. The drug loading content (DLC) was calculated from the mass of incorporated DOX divided by the weight of polymer. The amount of DOX loaded in micelle was determined by the absorption at 485 nm using UV-Vis spectrometry (UV-530, Jasco, Tokyo, Japan). The DOX solutions of various concentrations were prepared, and the absorptions of the solutions were measured to obtain a calibration curve [34,35]. The particle size was determined by dynamic light scattering (DLS) at 25 °C using a Zetasizer 3000HSA (Malvern Instruments Ltd, U.K.) with an excitation of 633 nm illuminated at a fixed angle of 90°.

### 3.3. Cytotoxicity of PEG-PCL-PEG Micelle

The *in vitro* cytotoxicity of micelle was tested against human breast cancer cell lines: MCF-7 by a cell viability assay (MTT assay). MCF-7 cells were seeded in 96-well plate at a density of 5 × 10^3^ cells/well and were incubated at 37 °C under a humidified atmosphere containing 5% CO_2_ for 24 h before assay. After that, the cells were further incubated in media containing various concentrations of micelle. After 24 h, the medium was removed and washed with PBS. MTT solution was added to each well followed by 4 h of incubation at 37 °C. Subsequently, the medium was removed and violet crystals were solubilized with DMSO (200 μL). After shaking slowly twice for 5 s, the absorbance of each well was determined using a Multiskan Spectrum spectrophotometer (Thermo Electron Corporation, Waltham, MA, U.S.) at 570 nm and 630 nm. The cell viability (%) was calculated as the ratio of the number of surviving cells in micelle-treated samples to that of control.

### 3.4. Measurement of Nitric Oxide of PEG-PCL-PEG Micelle

RAW 264.7 macrophage cells were seeded in a 96-well plate (1 × 10^4^ cells/well) and incubated in 37 °C, 5% CO_2_ for 1 day. Micellar solutions (PEG-PCL-PEG and DOX-loaded PEG-PCL-PEG micelles) at various concentrations were added to the cells in a final volume of 0.2 mL. Accordingly, polymer concentration range is from 0.001 to 1.0 mg/mL, DOX concentrations from the micelle solutions were 78.5, 39.2, 7.85 and 0.000785 μg/mL, respectively. The supernatants were collected after 24 h and NO production was determined by Greiss reagent (1% sulfanilamide, 2.5% H_3_PO_4_, 0.1% naphthylethylenediamine dihydrochloride). Briefly, 100 μL of culture medium was added to 100 μL of Greiss reagent solution and incubated for 15 min. The absorbance was then measured at 540 nm. In the control experiments, macrophages were incubated in a lipopolysaccharides (LPS) solution (100 ng/mL) and a micelle-free medium. Moreover, total protein extract was determined by Micro BCA Protein Assay.

### 3.5. Measurement of ROS in Macrophage Cells of PEG-PCL-PEG Micelle

A fluorometric assay using intracellular oxidation of 2,7-dichlorohydrofluorescein diacetate (DCFH-DA) was performed [36]. Raw264.7 cells were seeded in 3.5 mm plates at a density of 3 × 10^5^ cells/plate and were incubated at 37 °C under a humidified atmosphere containing 5% CO_2_ for 24 h before assay. After that, the cells were further incubated in media containing various concentrations of micelle (1.0 and 0.5 mg/mL). Medium and H_2_O_2_ solution (1 mM) were used as controls. After 24 h, the medium was removed and washed with PBS. Then DCFH-DA solution (20 μM) was added to each plate followed by 30 min of incubation at 37 °C. Subsequently, the medium was removed and observed under fluorescent microscope (green filter with an excitation of 485 nm and an emission of 530 nm). After that cells were lysed with Triton 0.5% in PBS for 30 min, and aliquots were transferred to the black 96-well plate. Then the fluorescence of dichlorofluorescein (DCF), which is the oxidized product of DCFH-DA, was measured using the microplate spectrofluorometer. Data were expressed as the percentage of the ROS level in the control group.

### 3.6. In Vitro Hemolytic Test of PEG-PCL-PEG Micelle

The experimental procedure described here is an adjustment of standard F-756-00 [37], which is based on colorimetric detection of Drabkin's solution. 0.7 mL of micellar solutions (PEG-PCL-PEG and DOX-loaded PEG-PCL-PEG micelles) at various concentrations were incubated in 0.1 mL of rabbit red blood cells at 37 °C and for 3 h. To make sure fresh rabbit blood was used in the test, the hemoglobin in as-harvested plasma of rabbit blood was found to be less than 220 μg/mL, which is regarded as basal level in the hemolysis test. Following incubation, the solution was centrifuged at 3800 rpm for 15 min. To determine the supernatant hemoglobin, 0.75 mL of Drabkin's solution was added to 0.25 mL of supernatant and the sample was allowed to stand for 15 min. The amount of cyanmethemoglobin in the supernatant was measured by spectrophotometer (JASCO UV-530, Tokyo, Japan) at a wavelength of 540 nm and then compared to a standard curve (hemoglobin concentrations ranging from 0.003 to 1.2 mg/mL). The percent hemolysis refers to the hemoglobin concentration in the supernatant of a blood sample not treated with micelles to the obtained percentage of micelle-induced hemolysis. Additionally, the absorption of the micellar DOX was determined at 540 nm in order to eliminate the effect of absorption of DOX. Finally, saline solution and double distilled water were used as negative and positive control, respectively.

### 3.7. In Vitro Cytotoxicity of DOX-Loaded Micelle

The *in vitro* cytotoxicity of DOX-loaded micelle was tested against human breast cancer cell lines: MCF-7. The cell culture medium was composed of DMEM with 10% fetal bovine serum and antibiotic/antimycotic. The cell viability was determined by tetrazolium dye (MTT) assay. MCF-7 cells were seeded in 96-well plates at a density of 5 × 10^3^ cells/well and were incubated at 37 °C in a humidified atmosphere containing 5% CO2 for 24 h before assay. After that, the cells were further incubated in media containing DOX-loaded micelle (DOX concentration 10 μg/mL). The cytotoxic effect was determined at 2, 24, 48, 72 and 96 h. For the time-points of 72 and 96 h, after 48 h incubation, the medium containing micellar DOX was removed and culture plated was rinsed with PBS. After that, medium-free micellar DOX was added and incubated. A solution of media with unloaded micelle (placebo) was used as a control to be compared with results obtained from micellar DOX. After interval time, the medium was removed and washed with PBS. MTT solution was added to each well followed by 4 h of incubation at 37 °C. Subsequently, the medium was removed and violet crystals were solubilized with DMSO (200 μL). After mild shaking twice for 5 s, the absorbance of each well was determined using a Multiskan Spectrum spectrophotometer (Thermo Electron Corporation, Waltham, MA, U.S.) at 570 nm and 630 nm. The cell viability (%) was calculated as the ratio of the number of surviving cells in drug-treated samples to that of control.

### 3.8. Cellular Uptake of DOX-Loaded Micelle

The cells (MCF-7) were seeded in BD Falcon culture slides (1 × 10^5^ cells/chamber) and incubated for 24 h. The cells were incubated with DOX-loaded micelle (DOX concentration 10 μg/mL). After 2, 24 and 48 h, the medium was removed. Hoechst 33342 (2 μg/mL) was added and incubated for 30 min. The cells were washed with cold PBS twice, and then fixed with formalin solution for 30 min, the formalin was removed and PBS was added. The fluorescence images of cells were obtained using a confocal laser scanning microscope (FluoView FV300, Olympus).

### 3.9. In Vivo Acute Toxicity of PEG-PCL-PEG Micelle

Nine female ICR mice with average body weight of 25–30 g were used in this study (Laboratory Animals, National Taiwan University, Taiwan). The mice were housed in the animal facility under controlled environment settings (25 °C, 60% humidity). All *in-vivo* experiments were carried out in accordance with the ethical guidelines for Animal Care and Use Committee of Chung Yuan Christian University. For acute toxicity test, mice (*n* = 3) were anesthetized with a cocktail of Zoletil (0.01 mL/kg) and Xylazine (0.01 mL/kg) solutions and then micellar solution was intravenously administrated at a dose of 71.4 mg/kg. PBS was used as control. For multiple dosing tests, 71.4 mg/kg micellar solution was injected on day 0, 3 and 6. The body weight of the mice was then monitored for a test period of 14 days post-injection. On day 14, the animals were sacrificed and livers and kidneys were collected. Histological examinations of the organs were carried out after dissection, then abnormal fibrotic tissue and loosen morphology were counted as adverse reaction due to the injection of micelle observed under a microscope (Eclipse 50i, Nikon, Japan).

### 3.10. Biodistribution of DOX-Loaded PEG-PCL-PEG Micelle

The *in vivo* biodistribution of DOX-loaded micelle was measured using female Wistar rats weighing 250–300 g. 18 rats were randomly divided into 2 groups as follows: Group A with intravenous (*i.v.*) injection of the pristine DOX solution and Group B with *i.v.* injection of DOX-loaded micelle. Three rats were injected *i.v.* with DOX or DOX-loaded micelle at the equivalent DOX 5 mg/kg dose at 1, 4 and 8 h time point. At the end of each time point, animals were sacrificed and blood was collected. Heart, liver, lung, kidney and spleen were harvested and homogenized in 20 mM KH_2_PO. After centrifugation, the supernatant was collected and kept at −20 °C until analyzed. For HPLC analysis, 0.4 mL of supernatant was mixed with 0.4 ml CH_3_CN, vortexed for 30 s and centrifuged at 15000× g for 10 min. The supernatant was collected and the solvent was removed at 40 degree under a stream of N_2_. The remaining sample was mixed with 0.25 mL of HPLC mobile phase solution [methanol: 0.1 % acetic acid and 0.1% ammonium hydroxide (25%), pH = 4.0; ratio: 60:40], filtered and transferred to auto sampler vials containing limited-volume inserts (150 μL) [38]. A C-18 column was used and the mobile phase was delivered at a rate of 1 mL/min. Sample (50 μL) was injected and the column effluent was detected with a UV detector (λ_ex_ = 485 nm, λ_emx_ = 580 nm). Several concentrations of DOX in plasma were used to create the standard curve (concentration: 25 ng/mL–1,000 ng/mL).

### 3.11. Antitumor Activity of DOX-loaded PEG-PCL-PEG Micelle

Three 5 week-old female BALB/c mice (National Laboratory Animal Center, Taiwan) were randomly assigned each of the following groups: Group A: control, group B: single administration of free DOX, group C: multiple administrations of free DOX, group D: single administration of DOX-loaded micelle, and group E: multiple administrations of DOX-loaded micelle. After quarantine in Animal Laboratory at CYCU, bilateral oophorectomy was performed. Estradiol (50 μg/mouse) was injected one week after oophorectomy. MCF-7 cells were trypsinized and resuspended in PBS. 5 × 10^6^ cells/0.1 mL were inoculated into the mammary fat pad of mice using a 27-gauge syringe. When the tumor volume reached approximately 200 mm^3^, the mice were injected intravenously via the tail vein at a dose of 5mg/kg (DOX equivalent) at day 0, 3 and 6 with free DOX, DOX-loaded micelle and PBS solution, respectively. The tumor inhibition activity was assessed by the tumor volume (TV), which was calculated as TV = (width^2^ × length) × ½. The dimension of the tumor measured by a caliper and the total body weight was also measured simultaneously.

## 4. Conclusions

In this study, the biocompatible and non-toxic triblock copolymer micelle was prepared in a self assembly method. *In vitro* NO release from macrophages and hemolytic tests confirmed that the PEG-PCL-PEG micelle induced very minor NO and hemolysis. The *in vitro* cytotoxicity study demonstrated that the micelle was safe and low cytotoxic. The cellular uptake of DOX-loaded micelle in MCF-7 was different from that of free DOX and the optimal time for MTT assay was 48 h. Furthermore, mice treated with prepared micelle in multiple doses did not develop toxic effects or die during the entire period of acute toxicity test. *In vivo* results showed that multiple injections of DOX-loaded micelle could prolong the circulation time and increase the therapeutic efficacy of DOX.

## Figures and Tables

**Table 1.** Characteristics of prepared DOX-loaded PEG-PCL-PEG micelle.

Sample	*M*_n_*a*	CMC (mg/mL)	Micellar size (nm)*b*	Micellar size (nm)*c*	DLE (%)	DLC (%)
ECE	31,000	5.1 × 10^−4^	85.3 ±2.1	91.6 ±7.2	57	8.5

a M_n_ was calculated from ^1^H NMR spectra;

b Micelle before DOX-loading (average size with ten measurements);

c Micelle after DOX-loading

## References

[1] Rivera E., Valero V., Arun B., Royce M., Adinin R., Hoelzer K., Walters R., Wade J.L., Pusztai L., Hortobagyi G.N. (2003). Phase ii study of pegylated liposomal doxorubicin in combination with gemcitabine in patients with metastatic breast cancer. J. Clin. Oncol..

[2] Shah J.J., Orlowski R.Z., Thomas S.K. (2009). Role of combination bortezomib and pegylated liposomal doxorubicin in the management of relapsed and/or refractory multiple myeloma. Therapeut. Clin. Risk Manag..

[3] Swenson C.E., Bolcsak L.E., Batist G., Guthrie T.H.J., Tkaczuk K.H., Boxenbaum H., Welles L., Chow S.C., Bhamra R., Chaikin P. (2003). Pharmacokinetics of doxorubicin administered i.v. As myocet (tlc d-99; liposome-encapsulated doxorubicin citrate) compared with conventional doxorubicin when given in combination with cyclophosphamide in patients with metastatic breast cancer. Anti-Cancer Drugs.

[4] Cuong N.V., Hsieh M.F. (2009). Recent advances in pharmacokinetics of polymeric excipients used in nanosized anti-cancer drugs. Curr. Drug Metab..

[5] Wong H.L., Bendayan R., Rauth A.M., Xue H.Y., Babakhanian K., Wu X.Y. (2006). A mechanistic study of enhanced doxorubicin uptake and retention in multidrug resistant breast cancer cells using a polymer-lipid hybrid nanoparticle system. J. Pharmacol. Exp. Ther..

[6] Xiong X.-B., Ma Z., Lai R., Lavasanifar A. (2010). The therapeutic response to multifunctional polymeric nano-conjugates in the targeted cellular and subcellular delivery of doxorubicin. Biomaterials.

[7] Marchi N., Hallene K., Kight K., Cucullo L., Moddel G., Bingaman W., Dini G., Vezzani A., Janigro D. (2004). Significance of mdr1 and multiple drug resistance in refractory human epileptic brain. BMC Medicine.

[8] Zhang Z., Huey Lee S., Feng S.S. (2007). Folate-decorated poly(lactide-co-glycolide)-vitamin e tpgs nanoparticles for targeted drug delivery. Biomaterials.

[9] Sharma A.K., Zhang L., Li S., Kelly D.L., Alakhov V.Y., Batrakova E.V., Kabanov A.V. (2008). Prevention of mdr development in leukemia cells by micelle-forming polymeric surfactant. J. Contr. Release.

[10] Kim D., Lee E.S., Oh K.T., Gao Z.G., Bae Y.H. (2008). Doxorubicin-loaded polymeric micelle overcomes multidrug resistance of cancer by double-targeting folate receptor and early endosomal ph. Small.

[11] Jabr-Milane L.S., van Vlerken L.E., Yadav S., Amiji M.M. (2008). Multi-functional nanocarriers to overcome tumor drug resistance. Cancer Treat. Rev..

[12] Blanco E., Kessinger C.W., Sumer B.D., Gao J. (2009). Multifunctional micellar nanomedicine for cancer therapy. Exp. Biol. Med..

[13] Torchilin V.P. (2006). Multifunctional nanocarriers. Adv. Drug Deliv. Rev..

[14] Maeda H., Wu J., Sawa T., Matsumura Y., Hori K. (2000). Tumor vascular permeability and the epr effect in macromolecular therapeutics: A review. J. Contr. Release.

[15] Arun K.I., Khaled G., Fang J., Maeda H. (2006). Exploiting the enhanced permeability and retention effect for tumor targeting. Drug Discov. Today.

[16] Cao N., Feng S.S. (2008). Doxorubicin conjugated to d-α-tocopheryl polyethylene glycol 1000 succinate (tpgs): Conjugation chemistry, characterization, *in vitro* and *in vivo* evaluation. Biomaterials.

[17] Sinha V.R., Bansal K., Kaushik R., Kumria R., Trehan A. (2004). Poly-ε-caprolactone microspheres and nanospheres: An overview. Int. J. Pharm..

[18] Otsuka H., Nagasaki Y., Kataoka K. (2003). Pegylated nanoparticles for biological and pharmaceutical applications. Adv. Drug Deliv. Rev..

[19] Shuai X., Merdan T., Unger F., Wittmar M., Kissel T. (2003). Novel biodegradable ternary copolymers hy-pei-g-pcl-b-peg: Synthesis, characterization, and potential as efficient nonviral gene delivery vectors. Macromolecules.

[20] Zahr A.S., Davis C.A., Pishko M.V. (2006). Macrophage uptake of core–shell nanoparticles surface modified with poly(ethylene glycol). Langmuir.

[21] Zhang Y., Zhuo R.X. (2005). Synthesis and in vitro drug release behavior of amphiphilic triblock copolymer nanoparticles based on poly (ethylene glycol) and polycaprolactone. Biomaterials.

[22] Gong C., We X., Wang X., Wang Y., Guo G., Mao Y., Luo F., Qian Z. (2010). Biodegradable self-assembled peg-pcl-peg micelles for hydrophobic honokiol delivery: I. Preparation and characterization. Nanotechnology.

[23] Xu B., Yuan J., Ding T., Gao Q. (2010). Amphiphilic biodegradable poly(ε-caprolactone)-poly(ethylene glycol)-poly(ε-caprolactone) triblock copolymers: Synthesis, characterization and their use as drug carriers for folic acid. Polym. Bull..

[24] Ge H., Hu Y., Jiang X., Cheng D., Yuan Y., Bi H., Yang C. (2002). Preparation, characterization, and drug release behaviors of drug nimodipine-loaded poly(ε-caprolactone)-poly(ethylene oxide)-poly(ε-caprolactone) amphiphilic triblock copolymer micelles. J. Pharm. Sci..

[25] Gou M., Zheng X., Men K., Zhang J., Zheng L., Wang X., Luo F., Zhao Y., Zhao X., Wei Y., Qian Z. (2009). Poly(ε-caprolactone)/poly(ethylene glycol)/poly(ε-caprolactone) nanoparticles: Preparation, characterization, and application in doxorubicin delivery. J. Phys. Chem. B.

[26] Cuong N.V., Hsieh M.F., Chen Y.T., Liau I. (2010). Doxorubicin-loaded nanosized micelles of a star-shaped poly(ε-caprolactone)-polyphosphoester block co-polymer for treatment of human breast cancer. J. Biomater. Sci. Polym. Ed..

[27] Cuong N.V., Hsieh M.F., Chen Y.T., Liau I. (2010). Synthesis and characterization of peg-pcl-peg triblock copolymers as carriers of doxorubicin for the treatment of breast cancer. J. Appl. Polym. Sci..

[28] Eom H.J., Choi J. (2009). Oxidative stress of silica nanoparticles in human bronchial epithelial cell, beas-2b. Toxicol. Vitro.

[29] Eliaz R.E., Nir S., Marty C., Szoka F.C. (2004). Determination and modeling of kinetics of cancer cell killing by doxorubicin and doxorubicin encapsulated in targeted liposomes. Cancer Res..

[30] Upadhyay K.K., Bhatt A.N., Mishra A.K., Dwarakanath B.S., Jain S., Schatz C., Le Meins J.-F., Farooque A., Chandraiah G., Jain A.K., Misra A., Lecommandoux S. (2010). The intracellular drug delivery and anti tumor activity of doxorubicin loaded poly(γ-benzyl l-glutamate)-b-hyaluronan polymersomes. Biomaterials.

[31] Liu S.Q., Wiradharma N., Gao S.J., Tong Y.W., Yang Y.Y. (2007). Bio-functional micelles self-assembled from a folate-conjugated block copolymer for targeted intracellular delivery of anticancer drugs. Biomaterials.

[32] Zhao H., Yung L.Y.L. (2008). Selectivity of folate conjugated polymer micelles against different tumor cells. Int. J. Pharm..

[33] Nie S. (2010). Understanding and overcoming major barriers in cancer nanomedicine. Nanomedicine.

[34] Hsieh M.F., Cuong N.V., Chen C.H., Chen Y.T., Yeh J.M. (2008). Nano-sized micelles of block copolymers of methoxy poly(ethylene glycol)-poly(ε-caprolactone)-graft-2-hydroxyethyl cellulose for doxorubicin delivery. J. Nanosci. Nanotechnol..

[35] Aliabadi H.M., Mahmud A., Sharifabadi A.D., Lavasanifar A. (2005). Micelles of methoxy poly(ethylene oxide)-b-poly(ε-caprolactone) as vehicles for the solubilization and controlled delivery of cyclosporine a. J. Contr. Release.

[36] Hsieh M.F., Lin T.Y., Gau R.J., Chang H.T., Lo Y.L., Lai C.H. (2005). Biodegradable polymeric nanoparticles bearing stealth peg shell and lipophilic polyester core. J. Chin. Inst. Chem. Engrs..

[37] ASTM F756-00 (2000). Standard Practice for Assessment of Hemolytic Properties of Materials.

[38] Wei G., Xiao S., Si D., Liu C. (2008). Improved hplc method for doxorubicin quantification in rat plasma to study the pharmacokinetics of micelle-encapsulated and liposome-encapsulated doxorubicin formulations. Biomed. Chromatogr..

